# Limited contribution of compensatory mutations to MDR/RR-TB clustering in Hunan Province, China: a population-based whole-genome sequencing study

**DOI:** 10.1128/spectrum.02597-25

**Published:** 2026-03-03

**Authors:** Wencong He, Yunhong Tan, Zexuan Song, Hua Bai, Peilei Hu, Xichao Ou, Bing Zhao, Xiangyi Liu, Yanlin Zhao

**Affiliations:** 1 Department of Clinical Laboratory, Capital Medical University, Beijing Tongren Hospital 12517, Beijing, China; 2 Hunan Provincial Chest Hospital, Tuberculosis Control Institution of Hunan Province, Changsha, Hunan, China; 3 Department of Clinical Laboratory, Capital Medical University, Capital Institute of Pediatrics 12517, Beijing, China; 4 National Key Laboratory of Intelligent Tracking and Forecasting for Infectious Diseases, Chinese Center for Disease Control and Prevention, National Center for Tuberculosis Control and Prevention 12415, Beijing, China; Central Texas Veterans Health Care System, Temple, Texas, USA

**Keywords:** tuberculosis, MDR/RR-TB, compensatory mutations, whole-genome sequencing, transmission

## Abstract

Transmission of rifampicin-resistant and multidrug-resistant tuberculosis (MDR/RR-TB) is a major driver of the global drug-resistant TB epidemic, yet the contribution of compensatory mutations to its spread remains uncertain. We analyzed 206 non-duplicate MDR/RR-TB isolates collected from five surveillance sites in Hunan Province, China, between 2013 and 2020 using whole-genome sequencing. Transmission clusters were defined by ≤12 single-nucleotide polymorphism (SNP) differences, and compensatory mutations in rpoA, rpoB, and rpoC were identified through a curated mutation catalog and phylogenetic evidence. Clusters were further classified based on whether compensatory mutations predated transmission (C-type), arose after transmission (M-type), or were absent (N-type), with M-type clusters excluded to reduce confounding. Among the 206 isolates, 63 (30.6%) carried at least one compensatory mutation, predominantly in rpoC, and 27 clusters (32.5%) were identified, ranging from 2 to 6 isolates. No significant difference was observed in clustering frequency between compensated and non-compensated strains (21.7% vs 32.8%, *P* = 0.113), nor in cluster size between C-type and N-type clusters (*P* = 0.961). These findings demonstrate that compe nsatory mutations do not significantly affect transmission clustering at the population level; instead, prolonged infectious periods associated with diagnostic delays and treatment challenges are more likely to sustain MDR/RR-TB spread, underscoring the importance of rapid detection, timely effective therapy, and robust patient management to curb transmission.

**IMPORTANCE:**

Understanding the drivers of multidrug-resistant and rifampicin-resistant tuberculosis (MDR/RR-TB) transmission is critical for global TB control. Compensatory mutations in RNA polymerase subunits have been proposed as key contributors to the success of drug-resistant strains, yet population-level evidence has been limited. In this population-based genomic study of 206 MDR/RR-TB isolates collected over 8 years in Hunan Province, China, we systematically examined the association between compensatory mutations and transmission clustering. Our results show that compensatory mutations did not significantly increase clustering frequency or cluster size, indicating a limited impact on the spread of MDR/RR-TB in the community. These findings challenge the prevailing assumption that compensatory evolution is a major determinant of transmission success and provide robust genomic evidence to refine our understanding of drug-resistant TB epidemiology.

## INTRODUCTION

Tuberculosis (TB), caused by *Mycobacterium tuberculosis* (MTB), continues to rank among the deadliest global infectious diseases, with an estimated 10.8 million new cases and 1.25 million deaths in 2023 (1). The rise of drug-resistant TB (DR-TB), particularly multidrug-resistant or rifampicin-resistant TB (MDR/RR-TB), has posed a major challenge to global TB control efforts (2). In China, one of the 30 highest-TB-burden countries, approximately 741,000 new TB cases and 29,000 MDR/RR-TB cases were reported in 2023, highlighting the urgency of refining targeted control strategies to curb this public health crisis (1).

The control of MDR/RR-TB remains a major challenge due to its diagnostic complexity, limited treatment options, and low cure rates (3, 4). This challenge is compounded by accumulating evidence that MDR/RR-TB strains show a higher rate of genomic clustering, which serves as an indicator of recent transmission, than drug-susceptible strains (3–7). However, whether this seemingly enhanced transmissibility reflects true biological fitness or results from external factors remains unclear.

A widely discussed explanation involves compensatory mutations, primarily in *rpoA*, *rpoB,* and *rpoC*, which encode subunits of RNA polymerase (8, 9). These mutations are thought to mitigate the fitness cost associated with resistance-conferring mutations (notably in *rpoB*), thereby restoring bacterial replicative capacity and enhancing transmissibility (10). Experimental studies *in vitro* and *in vivo* support this hypothesis, demonstrating improved growth kinetics in strains harboring such mutations (2, 8, 11–13).

Nevertheless, this explanation remains controversial. In recent years, studies examining the role of compensatory mutations in MDR/RR-TB transmission have yielded inconsistent results (8–11, 14). While some report strong associations between specific mutations and increased fitness or genomic clustering, others have found no significant effect (6, 9, 10, 14, 15). These discrepancies may reflect differences in methodology, limited sample sizes, short observation periods, or insufficient integration of genomic and epidemiological data (14, 16).

Critically, most existing studies lack population-based, longitudinal genomic surveillance, limiting our understanding of MDR/RR-TB transmission dynamics in real-world settings (11, 17, 18). To address these limitations, we conducted a population-based study across five surveillance sites over an 8-year period in Hunan province, one of the high TB burden regions in China. The objectives of this study were to systematically characterize the prevalence and molecular diversity of compensatory mutations within the *rpoA*, *rpoB,* and *rpoC* genes among MDR/RR-TB isolates; and to elucidate the impact of compensatory mutations on the transmission potential of MDR/RR-TB strains using whole-genome sequencing. Collectively, our findings will provide a scientific foundation for precision TB control strategies tailored to MDR/RR-TB transmission dynamics.

## MATERIALS AND METHODS

### Selection of MDR/RR-TB strains

This retrospective study included all MTB isolates confirmed as MDR/RR-TB by whole-genome sequencing (WGS) from routine drug resistance surveillance work in Hunan Province, China, between January 2013 and December 2020. These isolates were derived from all sputum smear-positive, suspected pulmonary TB patients who visited locally designated hospitals or dispensaries at the five surveillance sites in Hunan province—Hecheng, Yongshun, Qidong, Taojiang, and Leiyang—established during China’s first national drug resistance survey (19). Sputum specimens were cultured in county-level laboratories and subsequently transferred to provincial and national reference laboratories for species identification, drug susceptibility testing, and whole-genome sequencing. For patients with multiple isolates, only the earliest was analyzed. In total, 206 non-duplicate MDR/RR-TB strains confirmed by WGS were included in the final analysis.

### Whole-genome sequencing and preliminary analysis

Genomic DNA of MTB isolates was extracted using the CTAB method and sequenced following standard protocols reported previously (3). Read quality was assessed with FastQC (v0.11.9), and low-quality reads were filtered using Trimmomatic (v0.38) with default parameters and a minimum Phred score of 20 (3, 20). Cleaned paired-end reads were mapped to the H37Rv reference genome (NC_000962.3) using BWA-MEM (v0.7.17). Single-nucleotide polymorphisms (SNPs) and small indels were identified using SAMtools (v1.3.1) and GATK (v3.8.0). Variants were retained if they had ≥10× coverage, a Phred score ≥20, and ≥75% allele frequency (3, 21). Repetitive and hypervariable regions (e.g., PE/PPE/PGRS genes, phage, and mobile elements) were excluded.

### Identification of putative compensatory mutations

Putative compensatory mutations were identified among rifampicin-genotypically resistant isolates using a two-step, literature-based strategy. First, we matched rpoA/rpoB/rpoC variants to published catalogs of putative compensatory mutations (14). Second, we classified as compensatory any nonsynonymous rpoA/rpoB/rpoC mutations that occurred exclusively in rifampicin-resistant isolates and showed ≥2 independent acquisitions on distinct phylogenetic branches (9). The final compensatory set was defined as the union of variants identified by these two approaches. MTB isolates carrying mutations known to confer rifampicin resistance were classified as RR-TB; those also harboring mutations associated with isoniazid resistance were designated as MDR-TB.

### Genotypic resistance profiling and cluster analysis

SNPs used for phylogenetic inference were filtered to polymorphic sites callable in ≥95% of isolates and were used to infer a maximum-likelihood tree in IQ-TREE (v1.6.10) with 1,000 bootstrap replicates (3). Trees were visualized in iTOL (https://itol.embl.de/) and Chiplot (https://www.chiplot.online/) (3). Pairwise genetic distances between isolates were computed using Snp-dists (v0.8.2). Isolates differing by ≤12 SNPs were assigned to the same cluster, suggesting recent transmission. Cluster size was defined as the number of MTB strains contained in the cluster (3). Lineages were assigned using Fast-lineage-caller (https://github.com/farhat-lab/fast-lineage-caller), and genotypic drug resistance was predicted with TB Profiler (v3.0.8) (https://jodyphelan.gitbook.io/tb-profiler/) (3, 21).

### Statistical analysis

Categorical variables were expressed as percentages (%), and group comparisons were performed using the Chi-square or Wilcoxon rank-sum test, as appropriate. Statistical analyses were conducted using R (v4.3.2), with a significance threshold of *P* < 0.05.

## RESULTS

### Description of the study population

**Fig 1:**
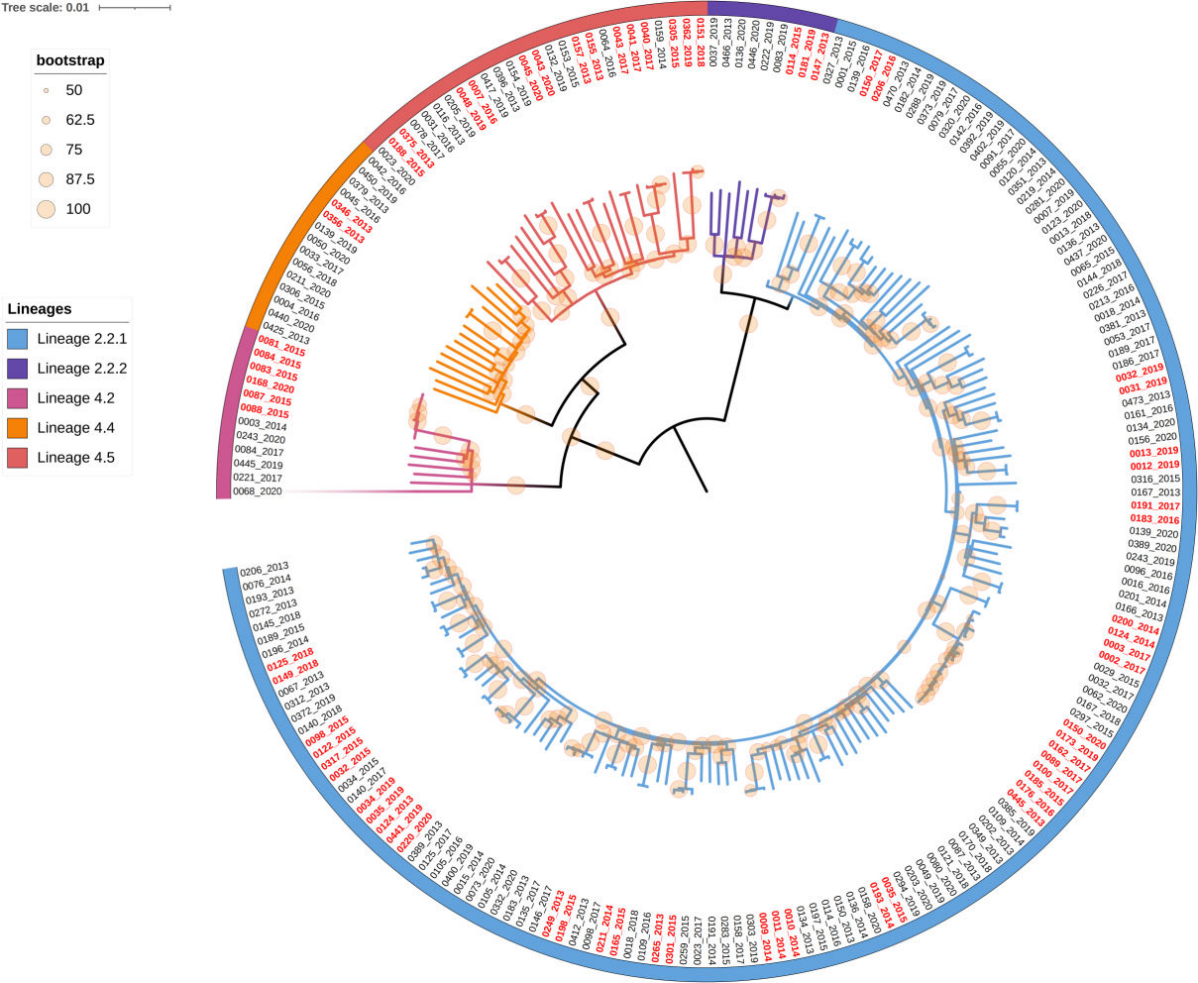
Phylogenetic tree of 206 MDR/RR-TB isolates. Note: branches in different colors represent distinct lineages and sub-lineages; red labels indicate genomic clusters identified using a genetic distance threshold of 12 SNPs.

From January 2013 to December 2020, 2,649 Mycobacterium tuberculosis isolates were collected from five drug resistance surveillance sites in Hunan Province and subjected to whole-genome sequencing. Among them, 206 isolates carried rifampicin resistance-associated mutations and were classified as genotypically MDR/RR-TB; these were included in the present study. Lineage 2.2.1 was the most prevalent among the MDR/RR-TB isolates (69.4%, 143/206), followed by Lineage 4.5 (12.6%, 26/206). Lineages 4.5 and 4.2 showed similar proportions, representing 7.3% (15/206) and 6.3% (13/206), respectively. Lineage 2.2.2 was the least common, accounting for only 4.4% (9/206). A total of 27 genomic clusters comprising 67 MDR/RR-TB isolates were identified based on a genetic distance threshold of 12 SNPs, corresponding to a clustering rate of 32.5% (67/206). Cluster sizes ranged from 2 to 6 isolates, with small clusters (2–3 isolates) being predominant, accounting for 82.1% (55/67) of all clustered strains (Fig. 1).

### Characteristics and distribution of putative compensatory mutations

**Fig 2:**
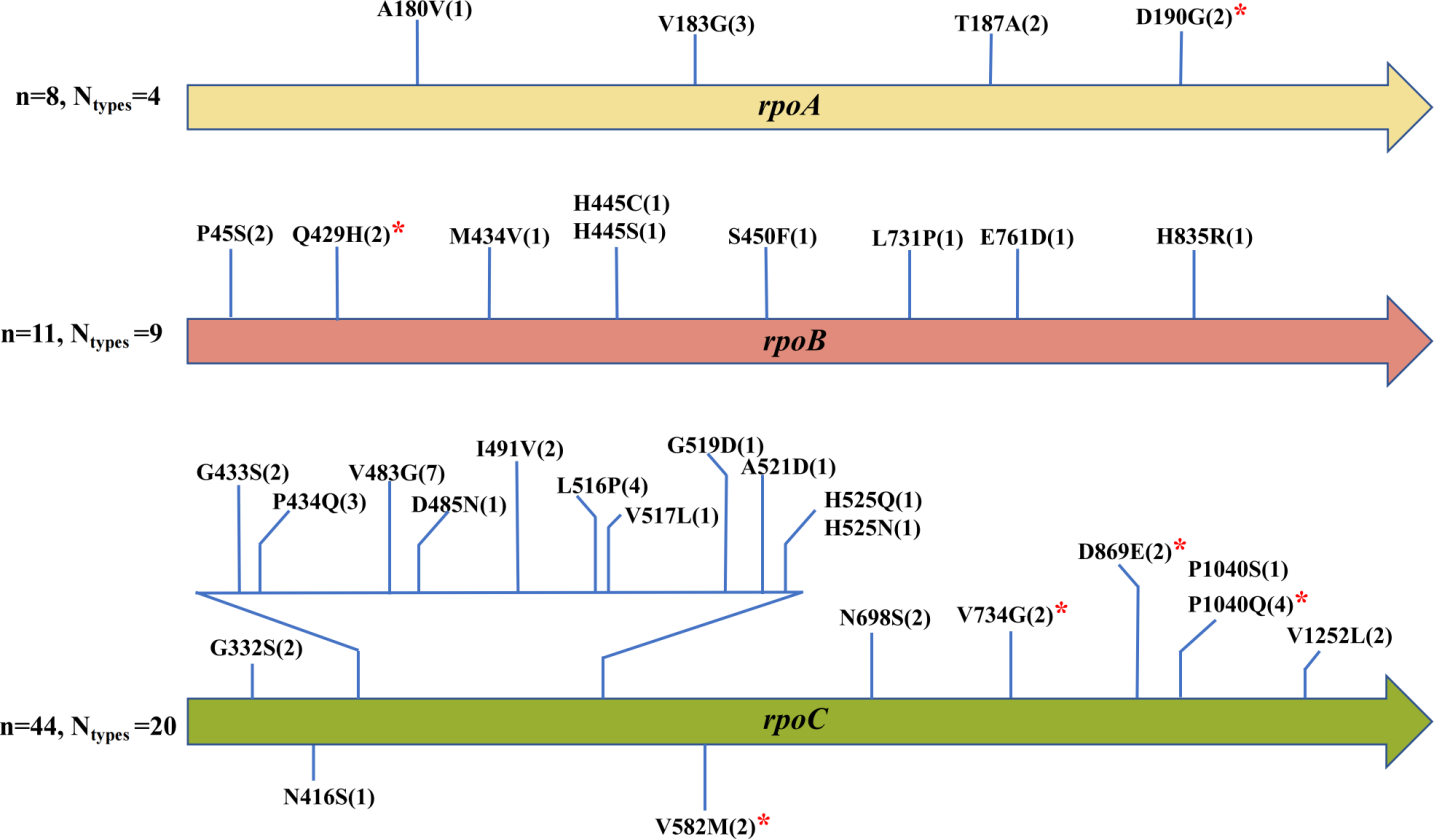
The distribution of putative compensatory mutations in the *rpoA*, *rpoB*, and *rpoC* genes detected in this study. Note: n indicates the number of isolates; N refers to the number of unique compensatory mutation types; values in parentheses show the number of isolates with each specific mutation type. Red asterisks indicate novel compensatory mutations identified in this study.

Among the 206 rifampicin genotypically resistant MTB isolates analyzed in this study, 63 (30.6%, 63/206) were found to harbor putative compensatory mutations, involving 33 distinct nonsynonymous variants, most of which were located in the *rpoC* gene. In addition, six previously unreported putative compensatory mutations (novel putative compensatory mutations) were identified, including one in *rpoA*, one in *rpoB*, and four in *rpoC*. The specific types and distributions of these putative compensatory mutations are shown in Fig. 2.

### Association analysis between compensatory mutations and genomic clustering of MDR/RR-TB strains

Compensatory mutations (CMs) may arise either before or after transmission events. The occurrence of CMs after transmission may confound the interpretation of their association with genomic clustering. To distinguish the timing of CM acquisition in relation to transmission, MDR/RR-TB clusters in this study were categorized into three types based on the presence and consistency of CMs within each cluster: (i) C-type clusters, in which all isolates carried the same CMs, suggesting that the mutations occurred prior to transmission; (ii) N-type clusters, in which none of the isolates carried CMs; and (iii) M-type clusters, in which only a subset of isolates carried CMs or different CMs were present within the same cluster, indicating that the mutations may have occurred after transmission.

**Fig 3:**
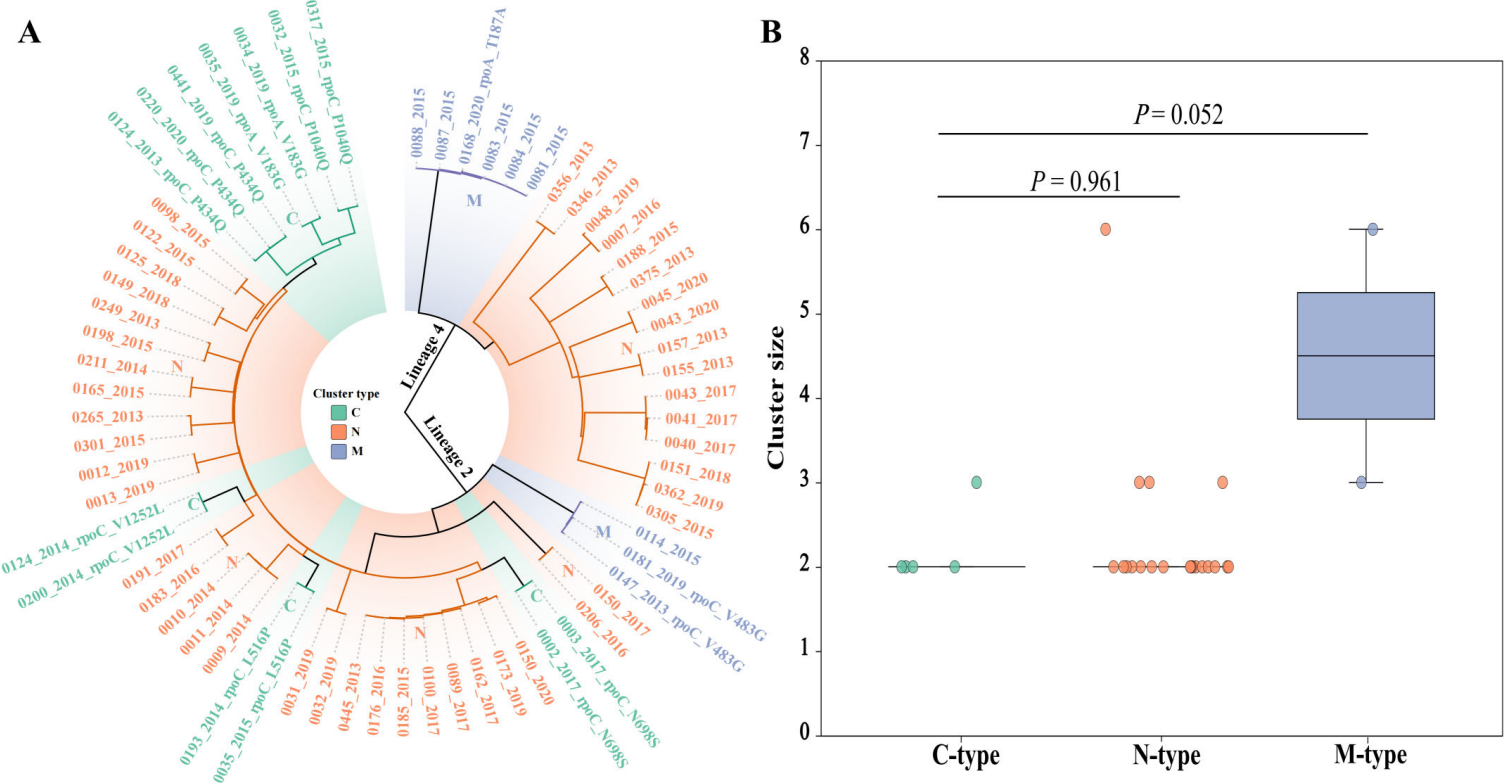
Association analysis between compensatory mutations and genomic clustering of MDR/RR-TB strains. Note: (**A**) Maximum likelihood phylogenetic tree of MDR/RR-TB clustered strains; (**B**) comparison of cluster sizes among three different strain types. The three colors represent different strain types: green for C-type, orange for N-type, and blue for M-type. *P* values were calculated using the Wilcoxon rank-sum test.

Among the 27 MDR/RR-TB genomic clusters identified in this study, 6 were classified as C-type, 2 as M-type, and 19 as N-type (Fig. 3A). Theoretically, if CMs promote the transmissibility of MDR/RR-TB strains, C-type clusters would be expected to be larger than N- or M-type clusters. However, no statistically significant difference in cluster size was observed between C-type and N-type clusters (*P* = 0.961) or between C-type and M-type clusters (*P* = 0.052) (Fig. 3B).

To further evaluate the overall association between CMs and genomic clustering, a comparative analysis was performed both with and without excluding M-type clusters. When M-type clusters were included, the clustering rate among strains with compensatory mutations was 25.4%, compared with 35.7% among those without compensatory mutations (χ² = 2.101, *P* = 0.147). When M-type clusters were excluded, the corresponding clustering rates were 21.7% and 32.8%, respectively (χ² = 2.511, *P* = 0.113), as shown in Table 1.

**TABLE 1:** Association analysis between compensatory mutations and clustering of MDR/RR-TB strains

Clustered MDR/RR-TB	Total	With CMs^*c*^ (%)	Without CMs (%)	χ^2^	*P*
Yes^*a*^	67	16 (25.4)	51 (35.7)	2.101	0.147
No	139	47 (74.6)	92 (64.3)		
Yes^*b*^	58	13 (21.7)	45 (32.8)	2.511	0.113
No	139	47 (78.3)	92 (67.2)		

^*a*^ Indicates clusters including M-type. CMs, compensatory mutations.

^*b*^ Indicates clusters excluding M-type.

^*c*^ CMs, compensatory mutations.

## DISCUSSION

Transmission of MDR/RR-TB results from a complex interplay of environmental conditions, host factors, and pathogen characteristics (22). Early mathematical models predicted a limited spread of MDR/RR-TB due to assumed fitness costs associated with resistance mutations (23). However, the global spread of MDR/RR-TB contradicts these predictions (1, 2). Accumulating evidence indicates that MDR/RR-TB strains exhibit higher transmission and clustering rates than drug-susceptible isolates, prompting investigation into the underlying mechanisms (3, 5, 7, 22). Two main hypotheses exist: compensatory mutations may restore fitness and enhance transmission (8, 10, 12, 24), or extended infectious periods—driven by diagnostic and treatment delays—may increase spread (9, 14). To clarify these drivers, we analyzed 8 years of MDR/RR-TB isolates from five surveillance sites in Hunan Province using WGS, aiming to elucidate the role of compensatory mutations in transmission dynamics.

Previous studies have suggested that MDR-TB strains harboring compensatory mutations are more likely to cluster (11, 17, 18). However, these studies treated compensatory mutations as a static feature, overlooking the possibility that such mutations may arise in secondary cases after transmission events, such as in M-type clusters (9). This oversight may partially confound the assessment of the association between compensatory mutations and strain clustering. Therefore, in our comparative analysis of the relationship between compensatory mutations and clustering, we excluded M-type clusters and focused only on C-type and N-type clusters. Our results showed no significant difference in clustering rates between strains with and without compensatory mutations. Additionally, the cluster sizes of C-type strains did not differ statistically from those of M-type or N-type clusters. These findings further suggest that compensatory mutations may not contribute substantially to the transmissibility of MDR/RR-TB strains. Importantly, our results are consistent with previous large-scale population-based studies showing that compensatory mutations exert only a limited effect on MDR/RR-TB transmission, demonstrated that C-type clusters were not larger than non-compensated clusters, while Chen et al. found no significant association between compensatory mutations and transmission across 17 global settings (9, 14). Together with our data, these findings suggest that compensatory mutations may restore bacterial fitness at the molecular level but rarely translate into measurable transmission advantages at the population level—particularly in regions with effective TB control measures. This highlights that the high transmission rate of MDR/RR-TB may be more attributable to prolonged infectious periods in the community due to delays in diagnosis and treatment (9). Therefore, enhancing early diagnosis, prompt initiation of treatment, and effective management of drug-resistant TB patients represent key strategies to curb the transmission of MDR/RR-TB.

A key strength of this study is the comprehensive inclusion of all culture-confirmed MDR/RR-TB isolates collected in the study region over an 8-year period. Utilizing this extensive data set, we conducted WGS-based clustering analysis based on SNP thresholds, which minimized potential misclassification due to incomplete sampling, limited surveillance duration, or suboptimal genomic resolution (3, 21). Additionally, building on the analytical framework proposed by Liu et al., we stratified compensatory mutations according to their likely timing—prior to versus following transmission—by differentiating C-type and M-type clusters (9). This refinement enhanced the precision of our interpretation regarding the role of compensatory evolution in transmission dynamics.

Nonetheless, several limitations should be acknowledged. First, isolates genetically related to strains outside the defined temporal or geographic scope may have been misclassified as non-clustered, potentially leading to underestimation of recent transmission. Second, the study was restricted to five national drug resistance surveillance sites in Hunan Province, which may limit the generalizability of our findings to other regions. Third, the predominance of small clusters (mostly consisting of two isolates) likely reflects effective infection control and timely case detection in the study region, which reduces opportunities for extended transmission chains. Consequently, the limited cluster sizes observed in this study may restrict the statistical power to detect subtle differences and may underestimate the potential contribution of compensatory mutations to transmission under higher-burden or less-controlled settings. Finally, the sample size, although regionally representative, remains modest. Future studies with broader geographic coverage and larger sample sizes are warranted to confirm and extend these findings.

In conclusion, our study revealed that compensatory mutations do not facilitate MDR/RR-TB transmission. The high spread of MDR/RR-TB is likely driven by extended infectious periods caused by diagnostic and therapeutic delays. Therefore, strengthening early detection, prompt initiation of therapy, and effective management of drug-resistant TB patients represents a crucial strategy for reducing MDR/RR-TB transmission.

## Data Availability

The sequencing data generated in this study have been submitted to the Genome Sequence Archive (CRA017099) at the National Genomics Data Center, China National Center for Bioinformation/Beijing Institute of Genomics, Chinese Academy of Sciences, and are publicly accessible at https://ngdc.cncb.ac.cn/gsa. Per-isolate accession numbers are provided in Table S1.

## SUPPLEMENTAL MATERIAL

Table S1 spectrum.02597-25-s0001.xlsx Sample IDs and corresponding GSA accession numbers (CRA017099) for 206 MDR/RR-TB strains included in this study.

ASM does not own the copyrights to Supplemental Material that may be linked to, or accessed through, an article. The authors have granted ASM a non-exclusive, world-wide license to publish the Supplemental Material files. Please contact the corresponding author directly for reuse.
